# Supplementary material for: Colloidal Lignin Particles as Adhesives for Soft Materials
Source: Nanomaterials (Basel). 2018 Dec 3;8(12):1001. doi: 10.3390/nano8121001 (PMC6315807; doi:10.3390/nano8121001)
Supplement: Supplementary file 1 [file nanomaterials-08-01001-s001.pdf]

Supplementary Material

Colloidal lignin particles as adhesives for soft materials

Maija-Liisa Mattinen,[1,*] Guillaume Riviere,[1] Alexander Henn,[1] Robertus Wahyu N. Nugroho,[1] Timo Leskinen,[1] Outi Nivala,[2] Juan José Valle-Delgado,[1] Mauri A. Kostiainen,[3] and Monika Österberg[1]

1. Adsorption of proteins on lignin surface

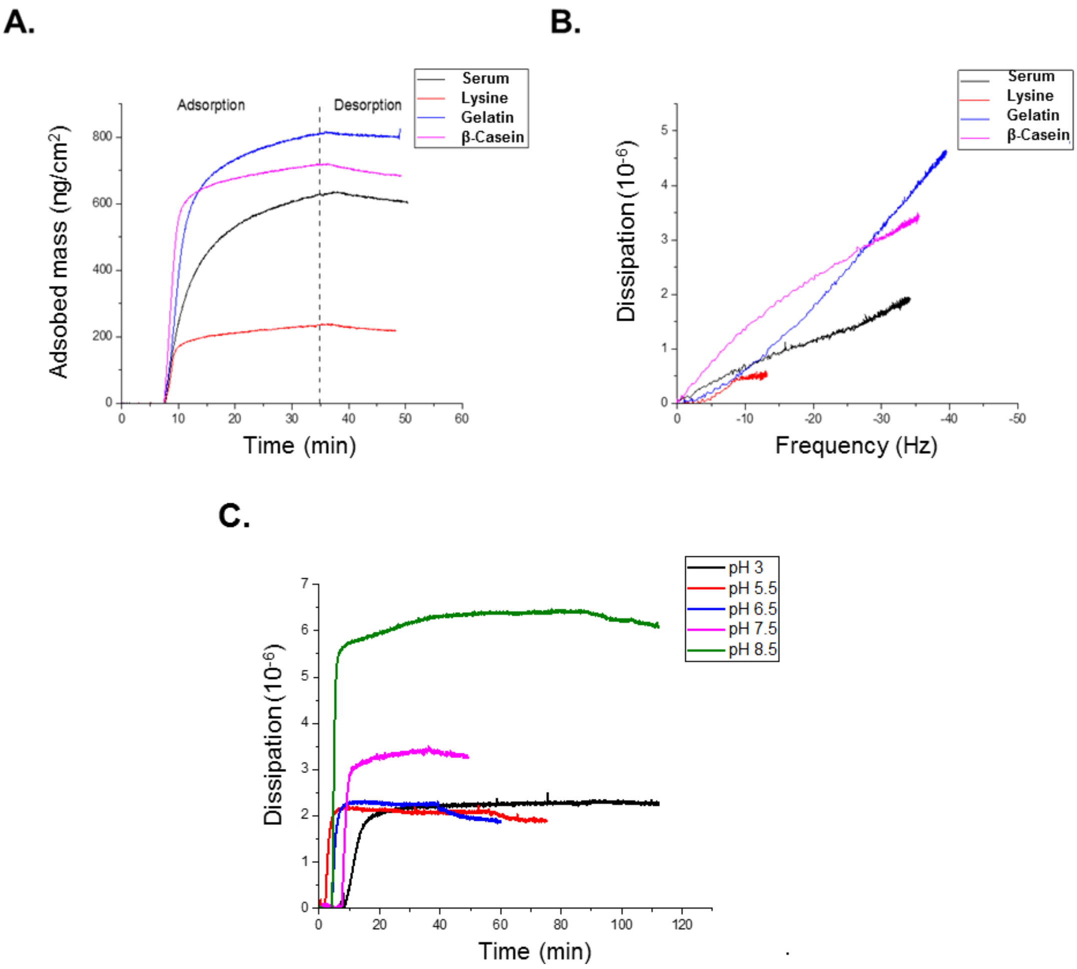

Figure S1. Adsorption of model proteins on lignin thin films analyzed by QCM-D. **A**) Frequency data (Δf5) from the measurement. **B**) Dissipation during the protein adsorption processes. **C**) Dissipation during the β-casein adsorption on lignin surface at different pH.

2. AFM images of CLPs coated with β-casein

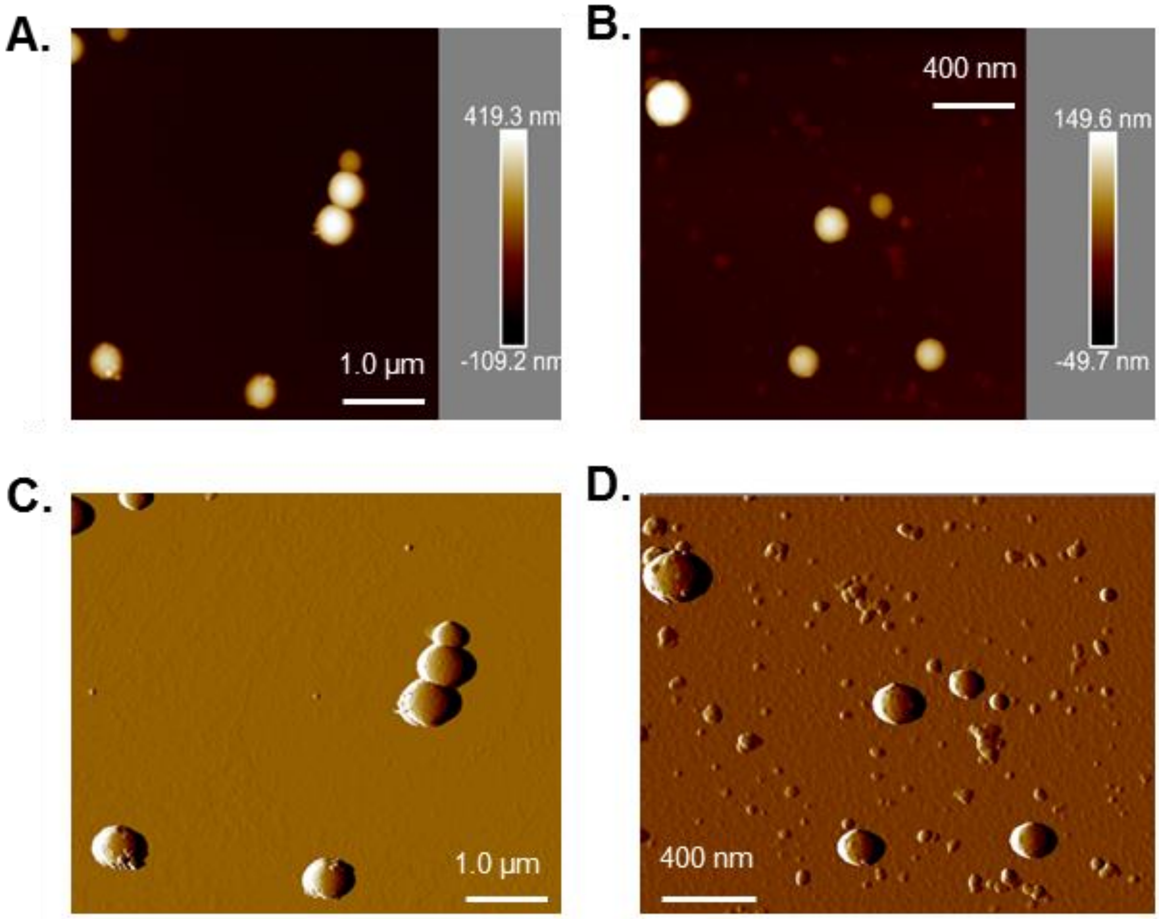

Figure S2. AFM images of β-casein coated CLPs. **A**) Reference (height mode). **B**) Protein coated CLPs (height mode). In (**C**) and (**D**) are shown the corresponding NPs in the amplitude mode.

3. Characterization of laccase treated lignins by FTIR

The selected bands areas (-OH, -C=O, aromatic, phenolic-OH) measured from the FTIR spectra were normalized with band at 1510 cm^{-1} corresponding to elongation vibration of -C=C- in aromatic compounds. The ratios of the selected band areas are shown in the Tables S1 to S3.

References

Table S1. A) Bands and regions used for drawing the baselines for the determination of band area. **B)** Area of the characteristic frequencies in the FTIR spectra and **C)** calculated ratio for the reference lignins dissolved at pH 4.0, 6.0 and 8.0.

A)

Band	Alcohol groups A	Carbonyl groups C	Aromatic groups L1	Aromatic groups L2	Phenolic groups P1	Phenolic group P2
**Region**	3669 – 3046	1803 – 1672	1537 – 1480	1477 – 1440	1439 – 1407	1238 – 1179
**Baseline**	3695 – 3039	1809 – 1546	1540 – 903	1544 – 903	1478 – 1398	1244 – 1169

B)

Area	Alcohol groups A	Carbonyl groups C	Aromatic groups L1	Aromatic groups L2	Phenolic groups P1	Phenolic group P2
**Lignoboost™ pH 4**	2.0	0.5	0.8	0.6	0.2	0.3
**Ref pH 6**	3.5	0.9	1.2	0.9	0.2	0.4
**Ref pH 8**	6.4	1.1	1.4	1.1	0.3	0.5

C)

Ratios	A/L1	A/L2	C/L1	C/L2	P1/L1	P1/L2	P2/L1	P2/L2
**Lignoboost™**	2.4	3.3	0.6	0.9	0.2	0.3	0.4	0.5
**pH 6 Ref**	2.8	3.8	0.8	1.0	0.2	0.3	0.3	0.5
**pH 8 Ref**	4.5	5.6	0.8	1.0	0.2	0.3	0.3	0.4

Expected trend
Unexpected trend

The results were considered unexpected when the variation was larger than ± 0.2 which is the most probably due to variation in the moisture content between the sample preparations.

pH 6

Table S2. A) Bands, regions and baselines used for the determination of the band area. **B)** Area of characteristic frequencies in the FTIR spectra and **C)** calculated ratio for the reference and treated-lignin at pH 6.

A)

**Band**	**Alcohol groups A**	**Carbonyl groups C**	**Aromatic groups L1**	**Aromatic groups L2**	**Phenolic groups P1**	**Phenolic group P2**
**Region**	3688 – 3046	1812 – 1678	1549 – 1479	1479 – 1443	1443 – 1398	1241 – 1173
**Baseline**	3685 – 3033	1812 – 901	1812 – 901	1812 – 901	1812 – 901	1812 – 901

B)

**Area**	**Alcohol groups A**	**Carbonyl groups C**	**Aromatic groups L1**	**Aromatic groups L2**	**Phenolic groups P1**	**Phenolic group P2**
**Ref pH 6**	3.5	0.9	1.3	0.9	1.0	2.6
**MaL pH 6**	3.9	1.0	1.2	0.9	1.0	2.5
**ThL pH 6**	1.9	0.6	0.6	0.4	0.5	1.2

C)

**Ratios**	**A/L1**	**A/L2**	**C/L1**	**C/L2**	**P1/L1**	**P1/L2**	**P2/L1**	**P2/L2**
**Ref pH 6**	2.8	4.0	0.7	1.0	0.8	1.1	2.0	2.9
**MaL pH 6**	3.2	4.4	0.8	1.1	0.8	1.1	2.1	2.9
**ThL pH 6**	3.4	4.5	1.0	1.3	0.8	1.1	2.1	2.8

Expected trend (green: C/L1, C/L2, P1/L2, P2/L2)

Unexpected trend (orange: A/L1, A/L2, P1/L1, P2/L1)

In general, the changes between the reference and laccase treated samples were minor. A/L were interfered by the residual moisture in the sample. P/L was expected as a decreasing trend while C/L was expected as an increasing trend.

pH 8

Table S3. A) Regions and the baselines used for the determination of area. **B)** Area of characteristic frequencies in the FTIR spectra and **C)** calculated ratio for the reference and treated-lignin at pH 8.

A)

**Band**	**Alcohol groups A**	**Carbonyl groups C**	**Aromatic groups L1**	**Aromatic groups L2**	**Phenolic groups P1**	**Phenolic group P2**
**Region**	3679 – 3045	1803 – 1678	1540 – 1478	1477 – 1440	1439 – 1397	1243 – 1174
**Baseline**	3684 – 3024	1812 – 901	1541 – 901	1544 – 902	1544 – 902	1544 – 902

B)

**Area**	**Alcohol groups A**	**Carbonyl groups C**	**Aromatic groups L1**	**Aromatic groups L2**	**Phenolic groups P1**	**Phenolic group P2**
**pH 8 Ref**	6.6	1.1	1.5	1.2	1.1	3.0
**pH 8 MaL**	5.7	1.5	1.7	1.3	1.2	3.5

C)

**Ratios**	**A/L1**	**A/L2**	**C/L1**	**C/L2**	**P1/L1**	**P1/L2**	**P2/L1**	**P2/L2**
**pH 8 Ref**	4.4	5.7	0.7	1.0	0.8	1.0	2.0	2.6
**pH 8 MaL**	3.4	4.4	0.9	1.1	0.7	0.9	2.1	2.7

Expected trend
Unexpected trend

Also, at pH 8 the differences between the references and laccase treated samples were minor. A/L and P/L were expected to degrease while C/L was expected to increase.

4. CLPs prepared from enzymatically oxidized Lignoboost™

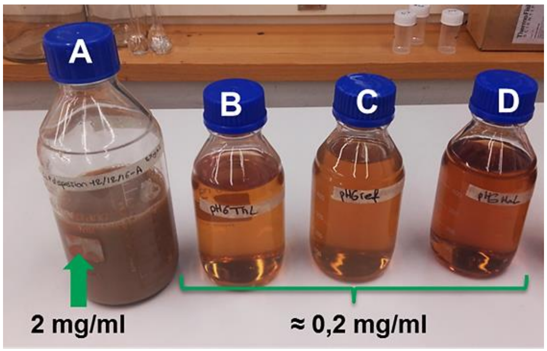

Figure S3. CLP dispersions prepared from laccases treated lignins. **A**) Lignoboost^{TM} using method developed Lievonen[25], **B**) ThL-, **C**) reference and **D**) MaL-treated lignins at pH 6.0.

5. AFM images from tiny CLPs for CNF coating

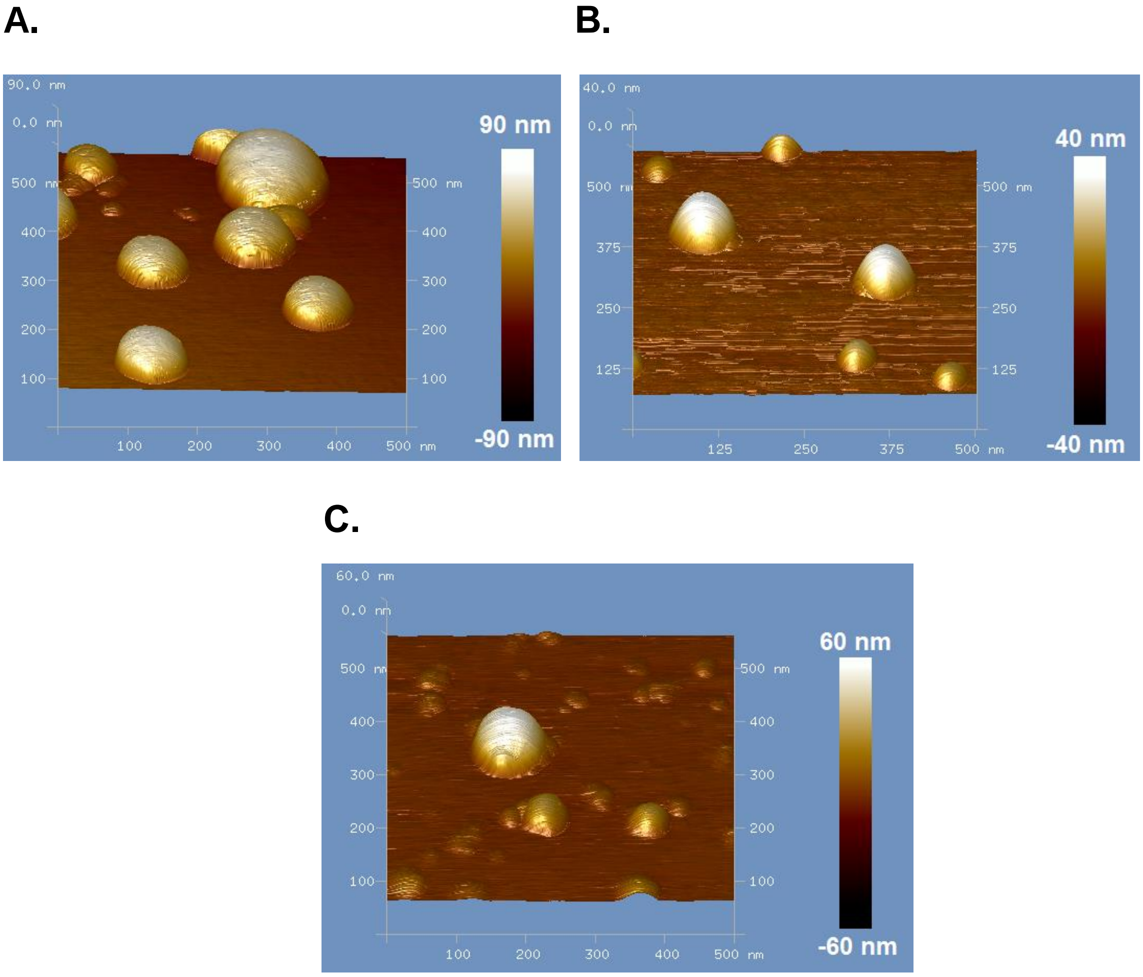

Figure S4. AFM height images from CLPs prepared from laccase treated lignin at pH 6. **A**) Reference, **B**) particles from MaL and **C**) ThL -treated lignin.

Table S4. Effect of time on the average particle size, zeta potential and polydispersity (PDI) of CLPs prepared from different lignins at starting concentration 0.5 g L^{-1}.

Days	Samples	Average size (nm)	Zeta potential (mV)	PDI
1	pH 6 Ref	120 ± 1	-34 ± 1	0.08 ± 0.01
	pH 6 MaL	117 ± 1	-35 ± 1	0.12 ± 0.02
	pH 6 ThL	120 ± 1	-33 ± 3	0.33 ± 0.03
	pH 8 Ref	107 ± 3	-30 ± 2	0.11 ± 0.00
	pH 8 MaL	83 ± 1	-37 ± 1	0.13 ± 0.02
4	pH 6 Ref	119 ± 1	-21 ± 1	0.09 ± 0.01
	pH 6 MaL	115 ± 1	-35 ± 1	0.11 ± 0.02
	pH 6 ThL	116 ± 1	-33 ± 2	0.30 ± 0.01
	pH 8 Ref	101 ± 1	-27 ± 1	0.10 ± 0.01
	pH 8 MaL	82 ± 1	-32 ± 1	0.13 0.01
7	pH 6 Ref	105 ± 1	-19 ± 1	0.12 ± 0.02
	pH 6 MaL	75 ± 1	-20 ± 2	0.13 ± 0.05
	pH 6 ThL	102 ± 2	-36 ± 3	0.37 ± 0.03
	pH 8 Ref	99 ± 1	-18 ± 1	0.11 ± 0.02
	pH 8 MaL	82 ± 2	-21 ± 1	0.13 ± 0.02
30	pH 6 Ref	120 ± 1	-26 ± 2	0.09 ± 0.01
	pH 6 MaL	121 ± 3	-37 ± 1	0.11 ± 0.02
	pH 6 ThL	121 ± 1	-39 ± 1	0.32 ± 0.02
	pH 8 Ref	104 ± 1	-36 ± 3	0.12 ± 0.02
	pH 8 MaL	83 ± 2	-33 ± 1	0.14± 0.01

6. TEM images from tiny CLPs used for CNF coating

A. **B.**

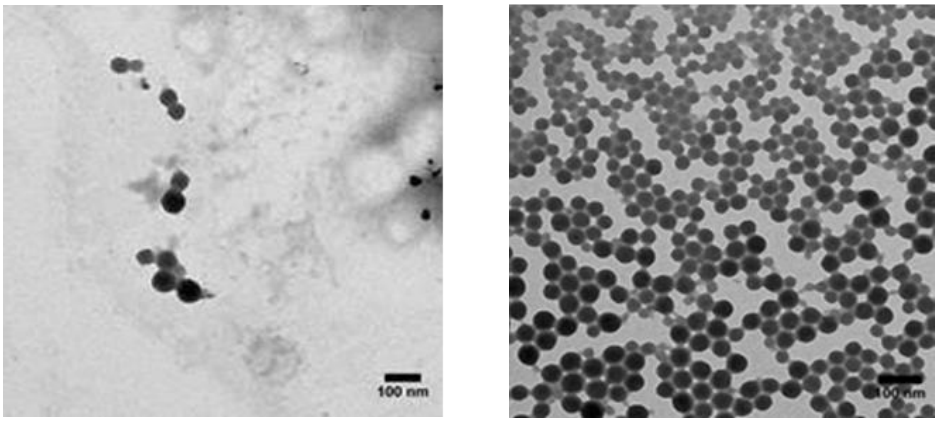

Figure S5. TEM images from CLPs prepared from enzymatically treated lignin one month after preparation. **A**) ThL-treatment (pH 6.0). **B**) Reference.

7. Coating poly(L-lysine) modified CLPs with poly(L-glutamic acid)

A.

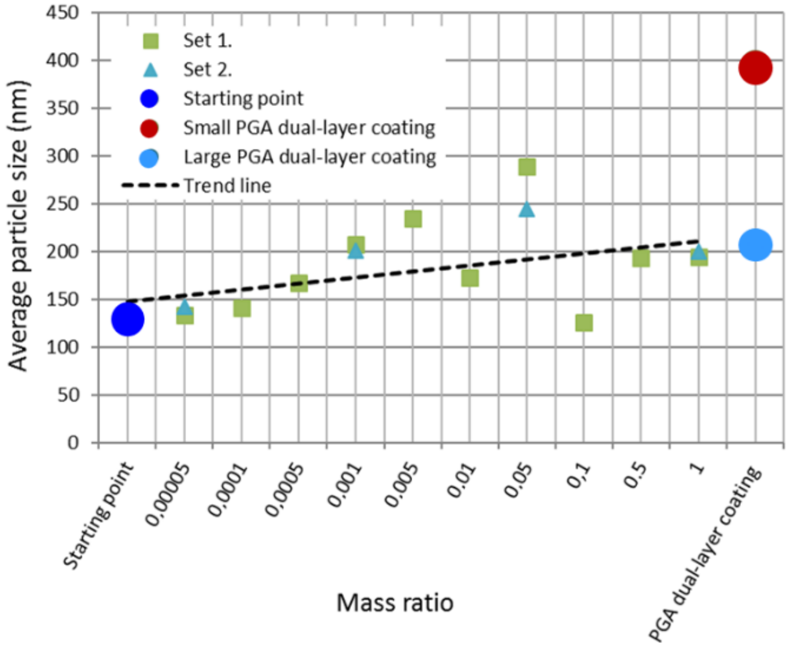

B.

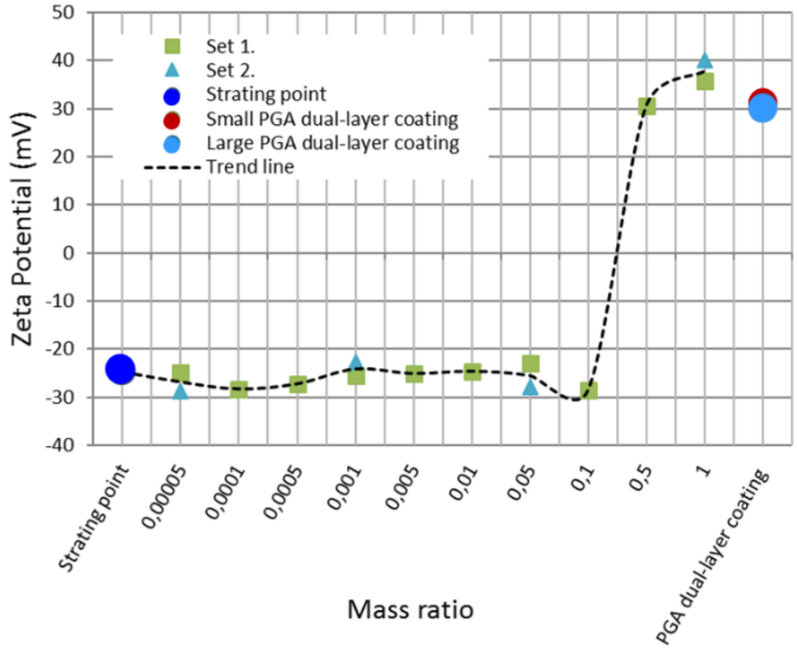

Figure S6. Average particle size and zeta potential of CLPs as a function of PL – CLP mass ratio. **A**) Average particle size (starting particle size: ca. 131 nm) and **B**) zeta potential. A trend line (a guide for the eye) is shown above the data points.

8. Adsorption of model proteins and CLPs on CNF surface

A.

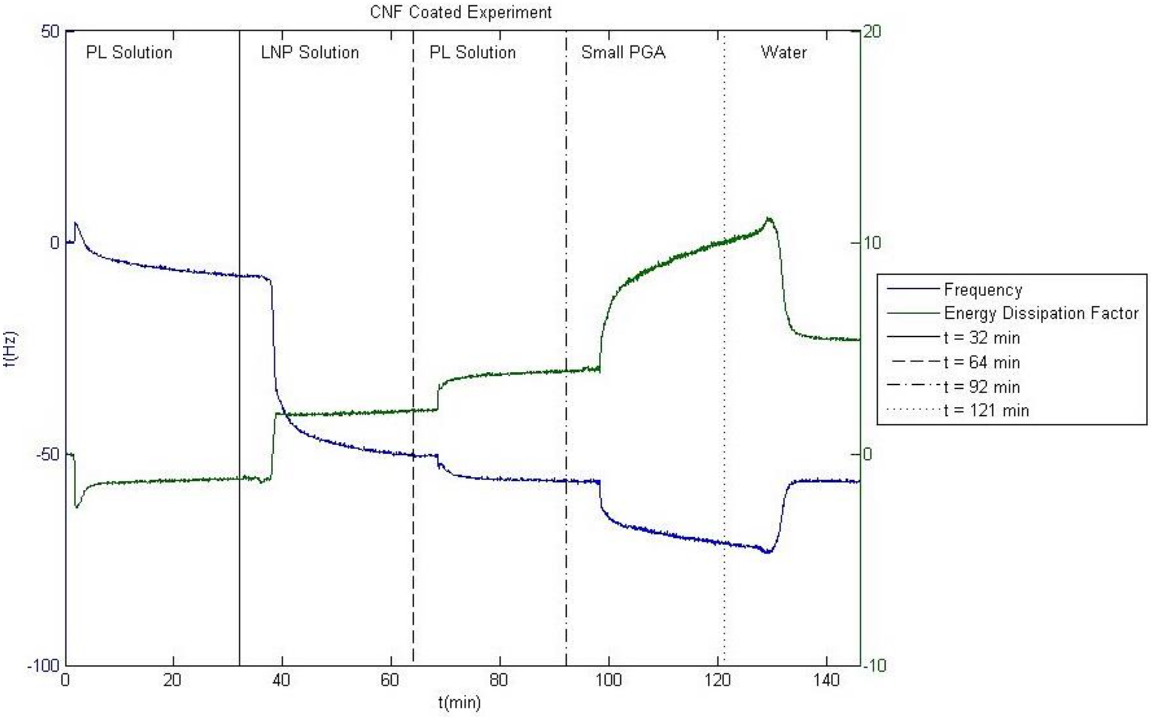

B.

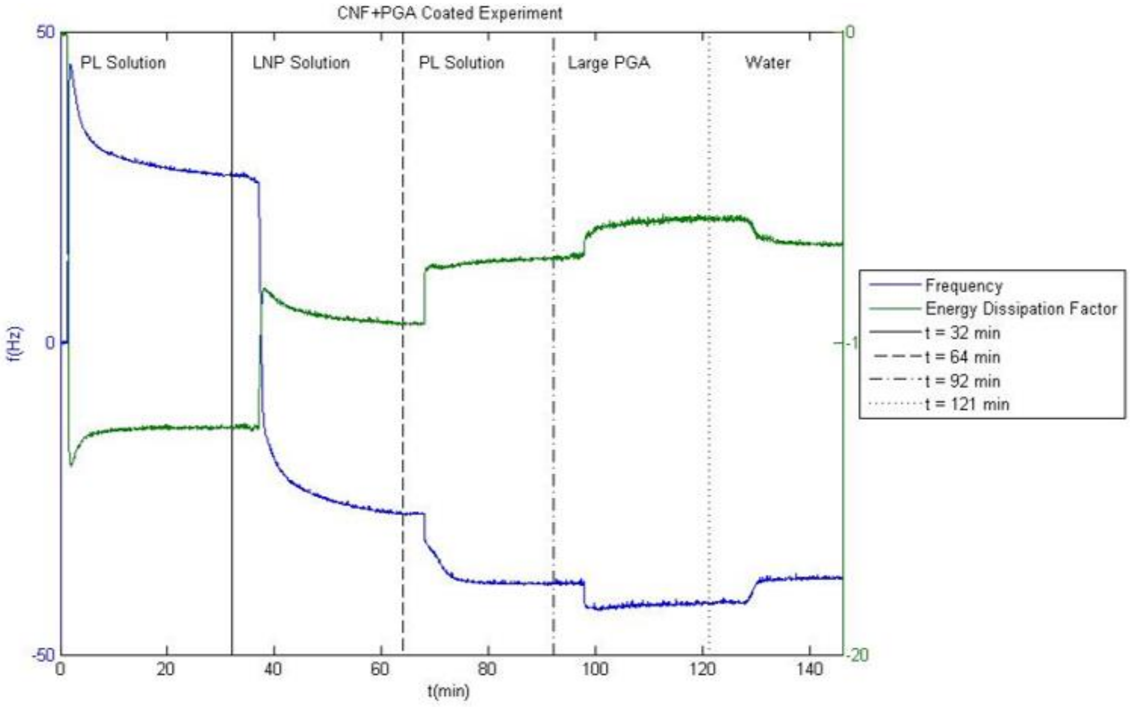

Figure S7. Adsorption of PL, PGA and CLPs on slightly negatively charged CNF analyzed by QCM-D. **A**) Small PGA (15 – 50 kDa) and **B**) large PGA (50 – 100 kDa).

9. Enzymatic stabilization of β-coated CLPs using Tgase

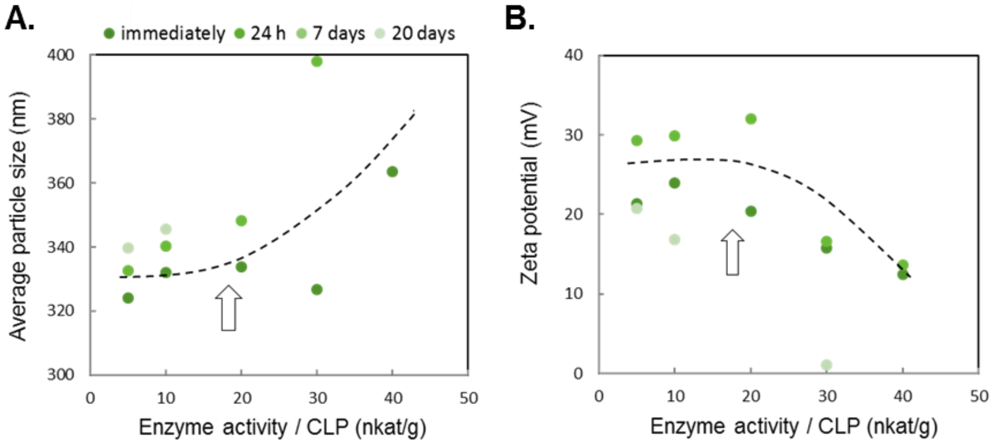

Figure S8. β-Casein coating and enzymatic stabilization of the particles with Tgase. Optimization of Tgase dosage for the cross-linking reactions as evidenced using **A**) particle size and **B**) zeta potential measurement as a function of time.

10. Inhibition of Tgase activity

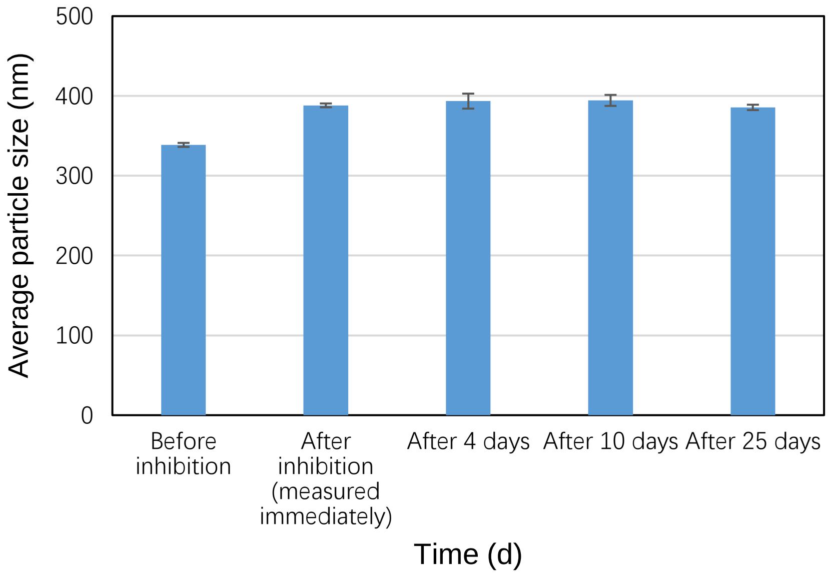

Figure S9. Variation of stabilized CLPs in size after removal of enzyme activity using ultracentrifugation.

11. Elasticity of β-casein coating on CLP cross-linked with Tgase

A.

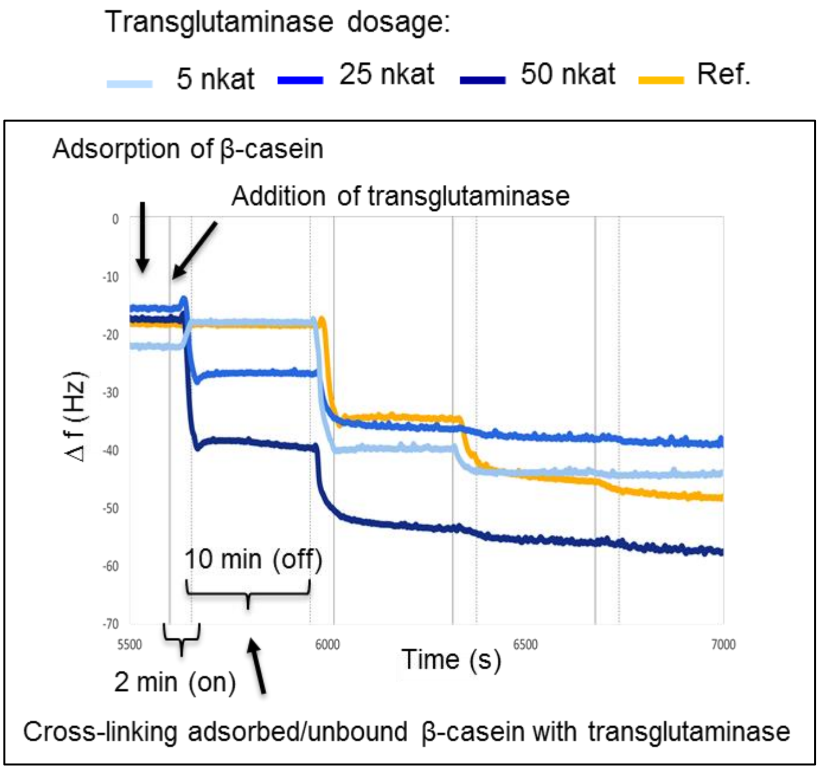

B.

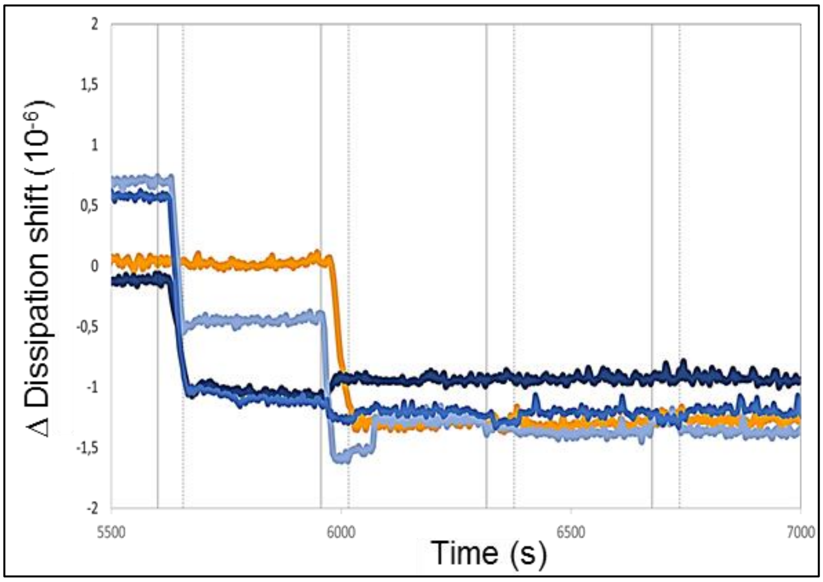

Figure S10. Elasticity of enzymatically cross-linked β-casein coating. Adsorption of β-casein on lignin surface following addition of Tgase in the QCM-D chamber and enzymatic cross-linking of adsorbed and unbound β-casein with Tgase using different enzyme dosages in the measuring cell (40 µl). In the reference Tgase activity was inhibited using heat treatment (60 °C).
